# Accuracy of virtual surgical planning in segmental osteotomy in combination with bimaxillary orthognathic surgery with surgery first approach

**DOI:** 10.1186/s12903-021-01892-7

**Published:** 2021-10-15

**Authors:** Xiaowu Ying, Kaiyue Tian, Kaiyu Zhang, Xiaohui Ma, Hongming Guo

**Affiliations:** 1 Department of Orthodontics, School of Stomatology, Capital Medical University, Beijing, 100050 China; 2 Department of Oral and Maxillofacial Plastic and Trauma, School of Stomatology, Capital Medical University, Beijing, China

**Keywords:** Surgery first approach, Virtual surgical planning, Accuracy, Dentofacial deformities

## Abstract

**Background:**

This study aimed to assess the accuracy of virtual surgical planning (VSP) in segmental osteotomy in combination with bimaxillary orthognathic surgery with surgery first approach (SFA) by means of three-dimensional (3D) measuring and superimposition, so as to promote the application of digital technology in combined orthodontic-orthognathic treatment.

**Methods:**

20 patients treated with segmental osteotomy in combination with bimaxillary orthognathic surgery with SFA from 2018 to 2020 were included. All of them acquired VSP performed by ProPlan CMF 3.0 software (Materialise Corporation, Belgium). The preoperative (T0) 3D model of VSP and the postoperative (T1) 3D model, reconstructed by the cone-beam computed tomography (CBCT) data acquired one week after surgery, were compared by measuring the 3D coordinates of the landmarks as well as 3D model superimposition for deviation analysis. The deviation analysis was achieved by Geomagic Studio 2013 (3D Systems Corporation, USA). The differences which represented the accuracy of VSP were evaluated by the root mean square deviation (RMSD) and the Bland–Altman method.

**Results:**

There was no statistically significant difference between the 3D coordinates of T1 and T0 (*P* > 0.05), and the mean overall RMSD was 1.37 mm, within the clinical relevance of 2 mm. The RMSD of sagittal direction (1.76 mm) was greater than that of coronal and vertical directions (1.09 mm and 1.24 mm), and the RMSD of maxillary and mandibular aspects were basically equal (1.30 mm and 1.45 mm). The Bland–Altman method showed the T0 and T1 measurements were in good agreement. The mean RMSD obtained from the deviation analysis was 1.85 mm, within the clinical relevance.

**Conclusions:**

VSP in segmental osteotomy in combination with bimaxillary orthognathic surgery with SFA proved to acquire accurate outcome in this study.

## Background

Severe dentofacial deformities usually require combined orthodontic- orthognathic treatment. Patients’ facial appearance can get improved in the very early stage with surgery first approach (SFA), where preoperative orthodontic treatment is removed or limited to no more than 2 months. The overall treatment time is shortened significantly accompanied by patients’ great satisfaction [1–3]. However, the postoperative occlusal relationship is not ideal so that the jaw stability needs to be improved [4]. Therefore, whether the best position of jaws and occlusal relationship can be designed and transferred accurately to the operation during surgical planning is of great significance.

Traditional surgical planning includes cephalometric analysis and operation simulation by cephalometric tracings and plaster model surgery [5]. There are inevitably deviations in the steps of dental cast making, face bow transferring, model surgery and so on, and the prediction of postoperative facial appearance is not intuitive enough [6–8]. With the development of digital imaging, computer-aided design and manufacturing (CAD/CAM) and three-dimensional (3D) printing technology, preoperative virtual surgical planning (VSP), 3D printing of surgical splints and evaluation of the surgery can all be achieved by computer software [9, 10]. Compared with the traditional method, 3D printing is more accurate, repeatable and time-saving [11]. Geert Van Hemelen et al. [12] found the accuracy of 3D virtual planning in hard tissue prediction was equivalent to traditional two-dimensional planning, which is better in soft tissue prediction. Zhang Nan et al. [13] and Jung-Hoon Kim et al. [14] found VSP accurate by the comparison of planned and actual results. Ngoc Hieu Tran et al. [15] found accurate outcome of 3D planning applied in skeletal class III cases with SFA.

At present, the researches on the accuracy of VSP mainly focus on skeletal class III cases, while few researches involve skeletal class II cases and segmental osteotomy, especially with SFA. However, with the gradual maturity of SFA and segmental osteotomy, the application of SFA in skeletal class II and class I cases has gradually increased [16–18]. Moreover, due to the difficulty of operation and rapid postoperative changes, the accuracy of VSP in segmental osteotomy in combination with bimaxillary orthognathic surgery with SFA is of great importance, which may cause the prolongation of the treatment course and even affect the final outcomes if not ideal. Therefore, the purpose of this study is to assess the accuracy of VSP in segmental osteotomy in combination with bimaxillary orthognathic surgery with SFA by means of 3D measuring and superimposition for deviation analysis, so as to promote the application of digital technology in combined orthodontic-orthognathic treatment.

## Materials and methods

### Patients

20 patients who received segmental osteotomy in combination with bimaxillary orthognathic surgery with SFA at Beijing Stomatological Hospital, Capital Medical University, from 2018 to 2020 were included. This was a retrospective study using the existing radiographic materials.

The inclusion criteria included: 1) adults; 2) acquired VSP before surgery (T0), which was a total digital workflow including intraoral dental scanning, cone-beam computed tomography (CBCT) scanning, 3D reconstruction, surgical simulation, design of digital surgical splints and 3D printing of the splints; 3) CBCT data acquired one week after surgery (T1) was available; 4) segmental osteotomy in combination with bimaxillary orthognathic surgery with SFA.

The exclusion criteria included: 1) severe diabetes, immunodeficiency, history of bisphosphonate therapy or other severe systemic conditions which might affect the study; 2) cleft lip and palate; 3) history of craniofacial trauma or orthognathic surgery.

VSP and orthognathic surgery were performed by one experienced orthodontic-orthognathic group. According to the preliminary experiment, α = 0.05, 1 − β = 0.90, and the minimum sample size is 14.

### VSP and surgical phase

The preoperative CBCT data in Digital Imaging and Communications in Medicine (DICOM) format was imported into ProPlan CMF 3.0 (Materialise Corporation, Belgium) for 3D reconstruction, and the dentition was replaced by the intraoral dental scanning through superimposition. The 3D model was segmented and the segments were repositioned, setting up the new occlusion as a simulation of surgery. Then the digital surgical splints were designed and 3D printed. The median splint was for the guidance the repositioning of segmented maxilla and the final splint would decide the final position of the mandible. Surgery involved segmental LeFort I osteotomy, bilateral sagittal split ramus osteotomy (BSSRO), mandibular anterior subapical osteotomy and genioplasty. Maxillary and mandibular rigid internal fixation was performed using titanium plates and screws. Skeletal anchorage was also placed for postoperative elastic traction.

### Establish reference planes

The postoperative CBCT data in DICOM format was imported into ProPlan CMF 3.0, then the thresholds of bone and teeth were set separately to perform Segmentation, Region grow, Calculate and Boolean operation to obtain the postoperative combined 3D model of skull, maxilla, mandible and dentitions.

**Fig. 1:**
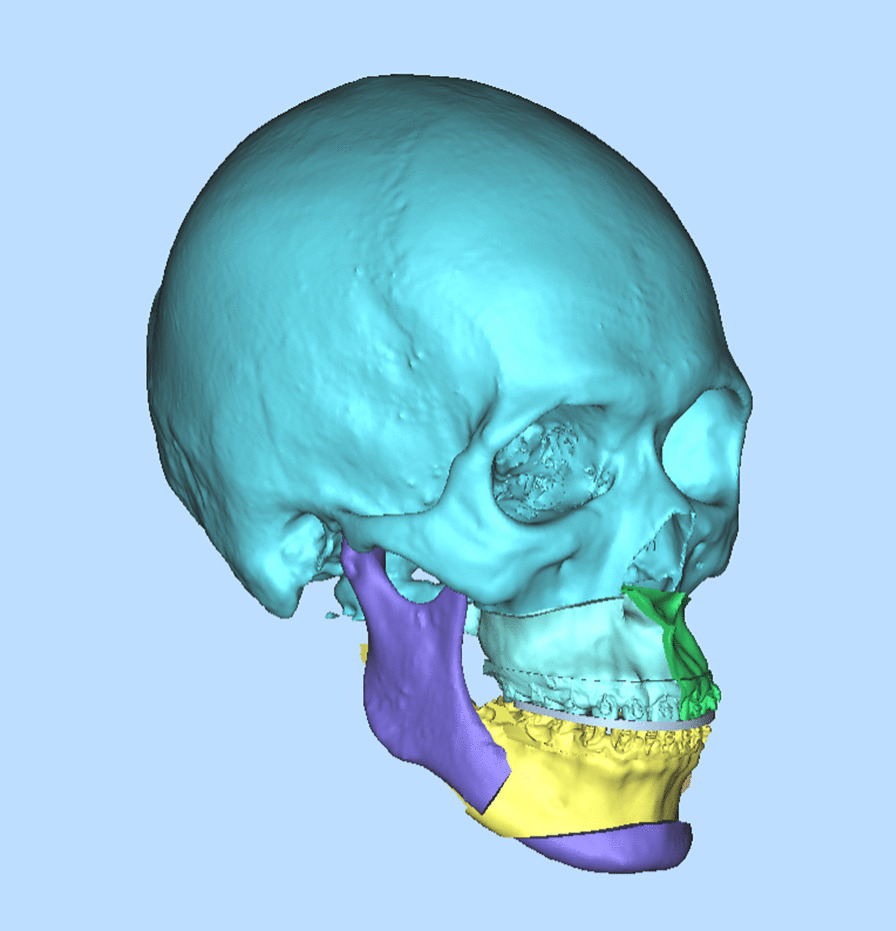
3D model of VSP

**Fig. 2:**
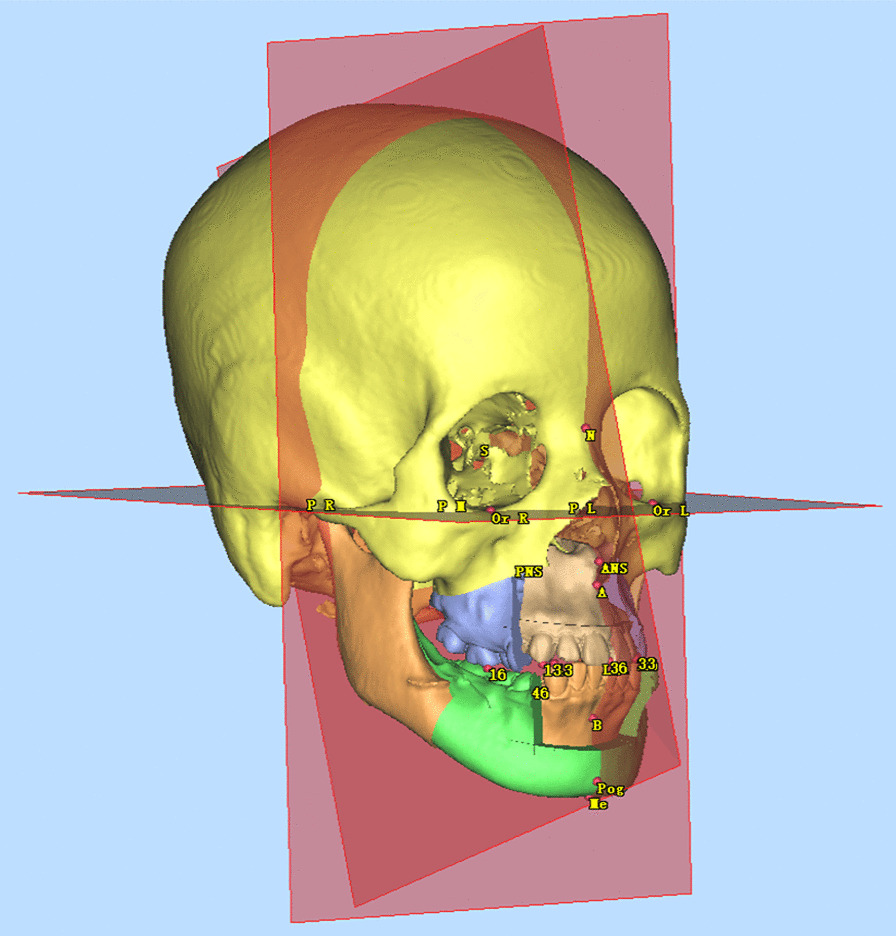
Reference planes

The VSP files in.sppc format were opened (Fig. 1), and custom planes were created in the Cephalometry section. The horizontal plane (HP) was constructed by the left orbitale (Or_L_), right orbitale (Or_R_) and the midpoint of the left and right porions (P_M_); the sagittal plane (SP) was perpendicular to HP through the nasion (N) and the sella point (S); the coronal plane (CP) was perpendicular to HP and SP through the sella point (Fig. 2). The same procedures were performed on the postoperative 3D model.

### Acquire the 3D coordinates of the landmarks

**Fig. 3:**
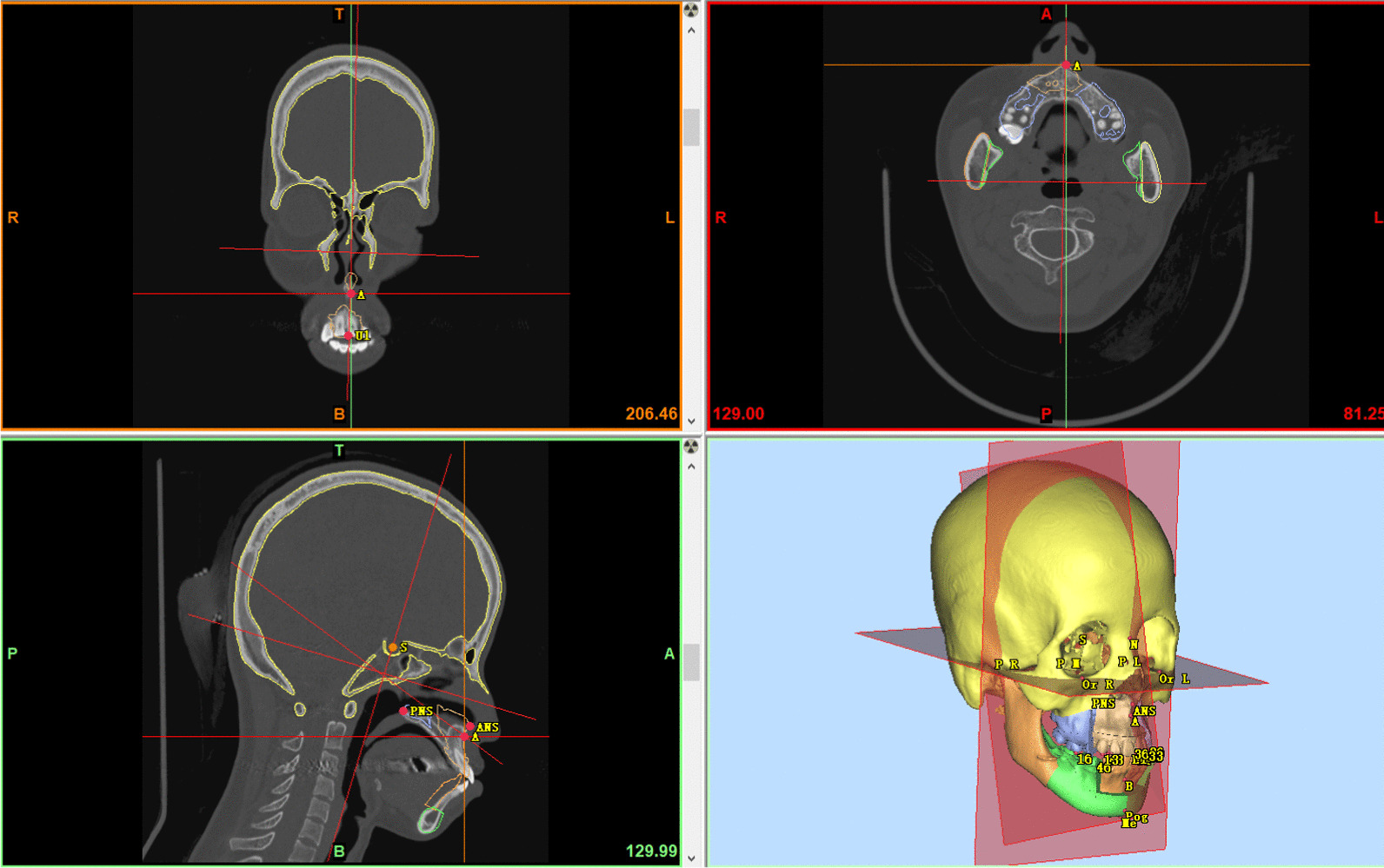
Locating the landmarks on multi-interfaces

The chosen landmarks below were firstly located on the 3D models then accurately adjusted on the CBCT sectional interfaces. The landmarks included anterior nasal spine (ANS), posterior nasal spine (PNS), subspinale (A), maxillary canine cusp (13, 23, 33, 43), supramental (B), contact point of maxillary central incisors (U1), contact point of mandibular central incisors (L1), mesiobuccal cusp of maxillary first molars (16, 26), mesiolingual cusp of mandibular first molars (36, 46), menton (Me) and pogonion (Pog). The distance from each point to HP was recorded as ‘z’, positive when below HP; the distance to SP was recorded as ‘x’, positive when on the left side of SP; the distance to CP was recorded as ‘y’, positive when in front of CP. The coordinate of each point was (x, y, z). Then the coordinates of the corresponding landmarks of T0 and T1 were compared (Fig. 3).

### 3D model superimposition and deviation analysis

**Fig. 4:**
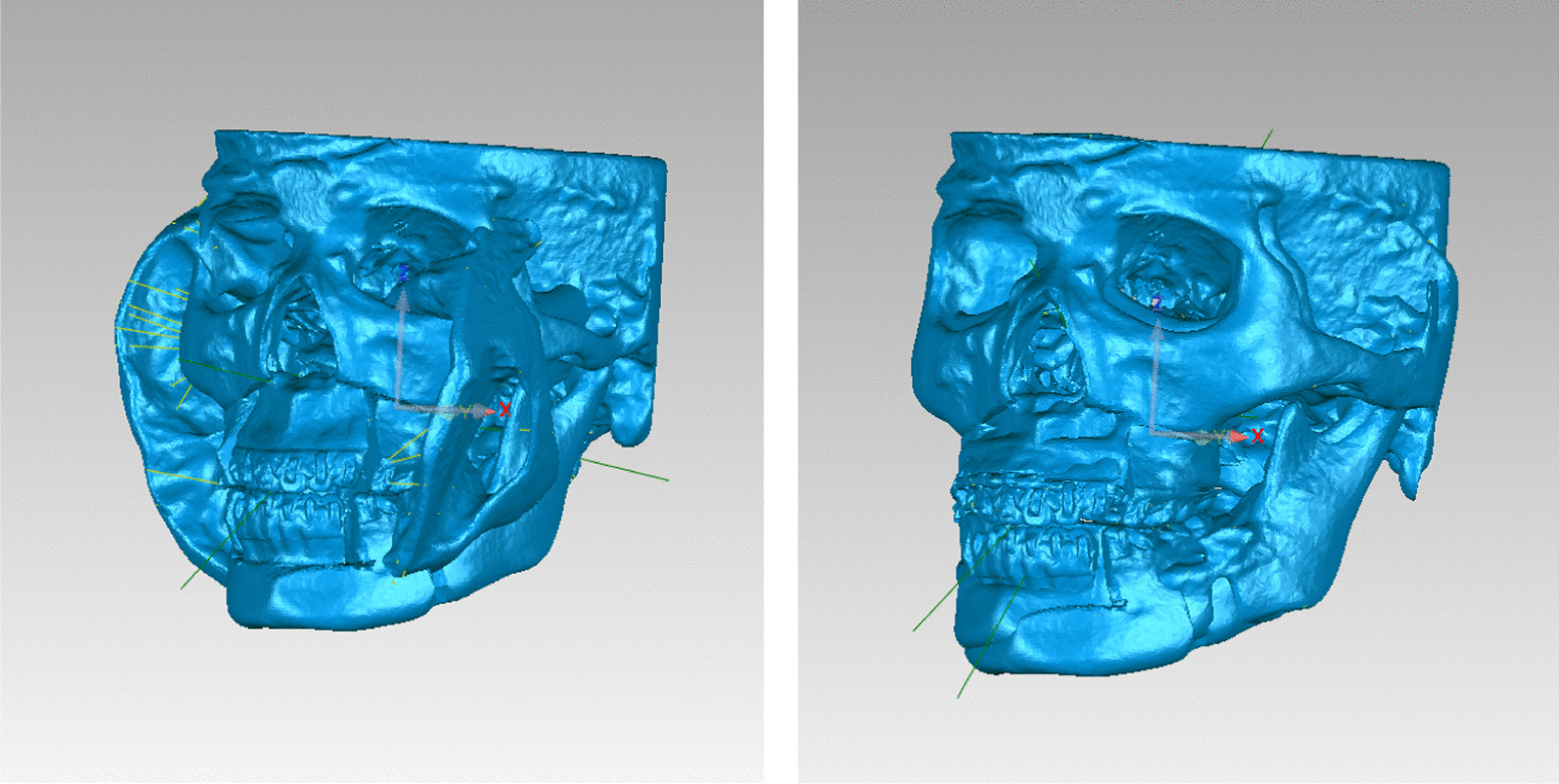
The process of best-fit superimposition

**Fig. 5:**
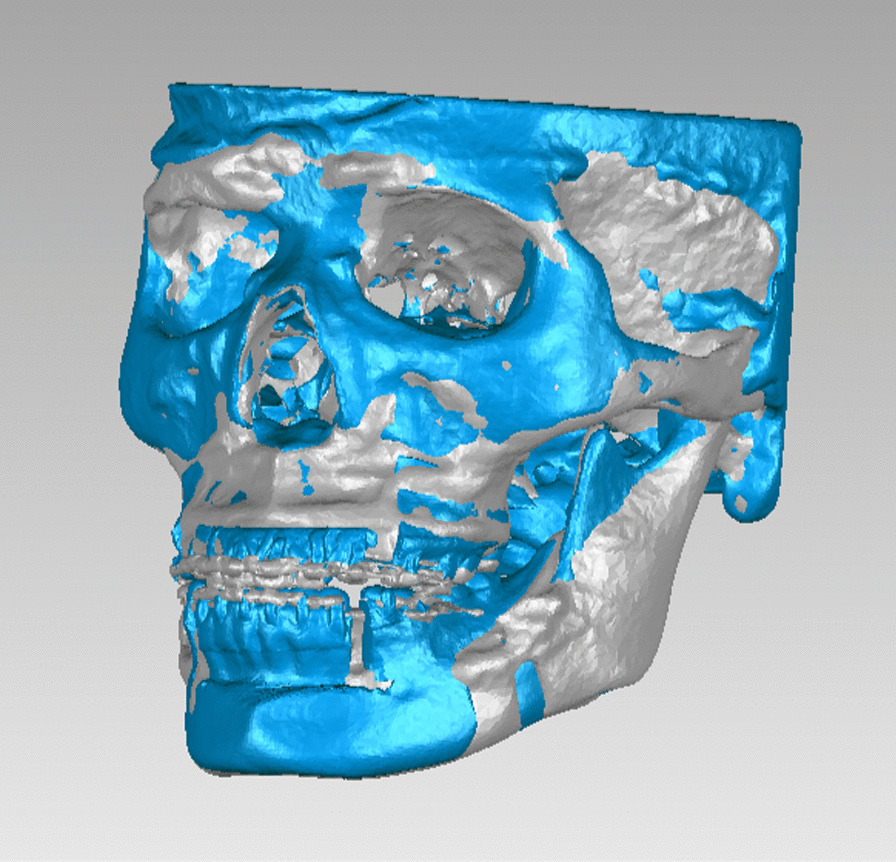
Best-fit superimposition was completed

The 3D models of T0 and T1 were exported to Geomagic Studio 2013 (3D Systems Corporation, USA) in.stl format, and the chins were both cut off according to the postoperative chin position for cases without guiding template of genioplasty, so as to eliminate the influence of chin on the overall data. Best-fit superimposition was performed on corresponding 3D models of T0 and T1 (Figs. 4, 5).

**Fig. 6:**
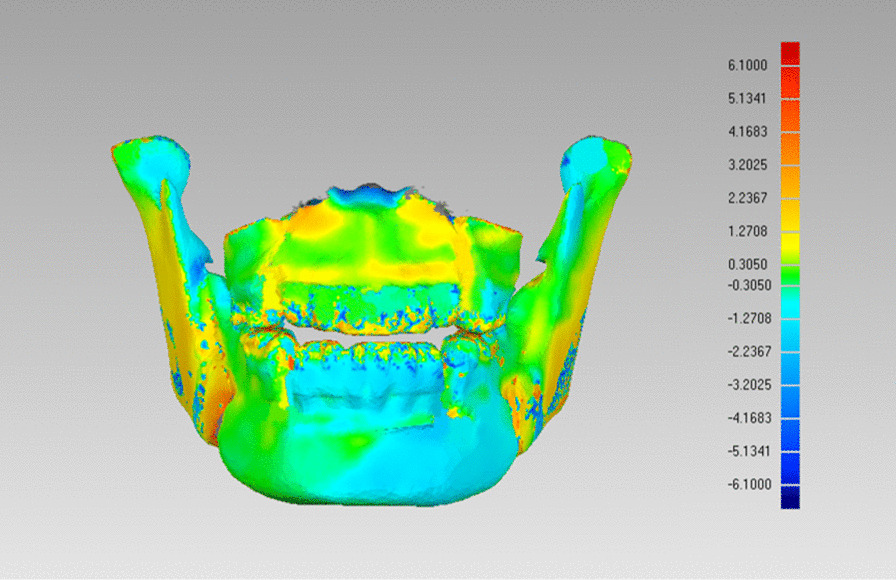
Chromatogram of deviation analysis

After the superimposition was completed, the software was used to automatically measure the euclidean distance between the 3D models and calculate the overall average, standard deviation and root mean square deviation (RMSD), that is, the deviation analysis, to describe the differences between the 3D models of T0 and T1 in the form of chromatograms and data (Fig. 6).

### Statistical analysis

All measurements were performed by the same person. Every measurement of the coordinate as well as the superimposition and deviation analysis of 3D models were performed 3 times, and the average was taken as the final value. SPSS 23.0 (IBM Corporation, USA) was used to test the normality of the sample through the Shapiro–Wilk test, then paired t test was used to analyze the difference between the coordinates of T0 and T1, 95% confidence interval, *P* < 0.05 was considered as a significant statistical difference; RMSD was used to evaluate the difference; Bland–Altman method was used to evaluate the consistency of the measurements.

## Results

16 women and 4 men were included, with a mean age of 25.00 ± 3.96 years (ranged from 18 to 33 years). 6 patients of skeletal class I bimaxillary protrusion (1.9° < ANB < 4.8°), 9 patients of skeletal class II (8.3° < ANB < 12.4°) and 5 patients of skeletal class III and mandibular deviation (− 1.6° < ANB < -4.6°) were diagnosed. 16 patients acquired segmental LeFort I osteotomy, bilateral sagittal split ramus osteotomy (BSSRO) and genioplasty (6 patients acquired mandibular anterior subapical osteotomy in the meantime); 3 patients acquired segmental LeFort I osteotomy, BSSRO and mandibular anterior subapical osteotomy; only 1 patient acquired segmental LeFort I osteotomy and BSSRO (Table 1).

**Table 1:** Sample demographic characteristics

Characteristics	
Sex (n)	
Male	4
Female	16
Age (years)	25 ± 3.96
Diagnosis (n) and ANB (°)	
Skeletal class I (6)	1.9 ~ 4.8
Skeletal class II (9)	8.3 ~ 12.4
Skeletal class III (5)	− 1.6 ~ − 4.6
Segmental LeFort I osteotomy and BSSRO	
With mandibular anterior subapical osteotomy and genioplasty (n)	6
With genioplasty (n)	10
With mandibular anterior subapical osteotomy (n)	3
None (n)	1

Because the guiding templates of genioplasty were absent, the measurements of chin landmarks ‘Pog’ and ‘Me’ only appeared in 4 cases without genioplasty to guarantee the reliability of the overall data. Therefore, the analyses below of overall data didn’t include ‘Pog’ and ‘Me’.

According to Table 2, the paired t-tests showed there was no statistically significant difference between the T0 and T1 measurements (*P* > 0.05). Except that the RMSD values of U1(y) and 23(y), 2.07 mm and 2.09 mm, were slightly larger than the clinical relevance of 2 mm [19–21], most RMSD values were within the clinical relevance. Therefore, the accuracy of VSP in this study was acceptable verified by 3D measuring (Table 2).

**Table 2:** Differences of landmark coordinates between VSP and actual result (mm)

Coordinates	T1 − T0		*P*	RMSD
	Mean	SD		
ANS x	0.08	0.66	0.71	0.63
y	− 0.65	1.76	0.27	1.79
z	0.34	1.40	0.46	1.37
PNS x	0.28	0.96	0.38	0.96
y	− 0.62	1.38	0.19	1.45
z	0.78	1.13	0.06	1.32
A x	0.18	0.68	0.27	0.69
y	− 0.32	1.40	0.32	1.16
z	0.13	1.05	0.60	1.03
U1 x	− 0.09	1.10	0.80	1.05
y	0.22	2.17	0.76	2.07
z	− 0.54	1.38	0.25	1.42
13 x	− 0.42	1.19	0.30	1.21
y	0.27	1.79	0.65	1.72
z	− 0.50	0.82	0.09	0.92
23 x	− 0.06	1.12	0.87	1.07
y	− 0.24	2.18	0.74	2.09
z	− 0.88	1.27	0.06	1.49
16 x	− 0.03	1.24	0.94	1.18
y	− 0.44	1.91	0.49	1.86
z	− 0.43	0.80	0.12	0.87
26 x	− 0.17	1.11	0.64	1.07
y	0.17	1.89	0.89	1.80
z	− 0.52	0.97	0.12	1.06
B x	− 0.43	1.35	0.34	1.35
y	− 0.11	1.74	0.85	1.65
z	0.09	1.21	0.82	1.15
L1 x	− 0.33	1.25	0.43	1.23
y	0.33	1.83	0.58	1.77
z	− 0.23	1.64	0.67	1.58
33 x	− 0.33	1.41	0.48	1.37
y	0.21	1.86	0.73	1.77
z	− 0.13	1.83	0.83	1.74
43 x	− 0.30	1.27	0.47	1.24
y	0.29	1.88	0.64	1.81
z	− 0.15	1.66	0.78	1.58
36 x	− 0.61	1.11	0.12	1.21
y	0.09	2.00	0.89	1.90
z	0.38	0.84	0.18	0.88
46 x	− 0.21	1.07	0.55	1.04
y	− 0.06	1.92	0.92	1.82
z	0.35	0.99	0.29	1.00
Me x	− 0.45	1.48	0.59	1.36
y	− 0.78	1.73	0.44	1.69
z	0.08	1.02	0.89	0.89
Pog x	− 0.78	0.57	0.07	0.92
y	− 0.15	0.90	0.76	0.79
z	0.20	1.06	0.73	0.94

*T1* postoperative actual result, *T0* preoperative VSP, *SD* standard deviation

The mean RMSD of the coronal direction (x) was 1.09 ± 0.22 mm, while that of the sagittal direction (y) and the vertical direction (z) were 1.76 ± 0.23 mm and 1.24 ± 0.29 mm. Moreover, U1(y) and 23(y) mentioned above were both in the sagittal direction. In consequence, the accuracy of VSP in this study was worse in sagittal direction than that in coronal and vertical directions (Table 3).

**Table 3:** Directional, maxillary and mandibular overall RMSD (mm)

	RMSD	
	Mean	SD
Coronal (x)	1.09	0.22
Sagittal (y)	1.76	0.23
Vertical (z)	1.24	0.29
Maxillary	1.30	0.41
Mandibular	1.45	0.32
Overall	1.37	0.38

*SD* standard deviation

The maxillary overall RMSD was 1.30 ± 0.41 mm while the mandibular overall RMSD was 1.45 ± 0.32 mm, which meant the accuracy of VSP in maxilla was slightly better than that in mandible. The overall mean RMSD was 1.37 ± 0.38 mm (Table 3).

**Fig. 7:**
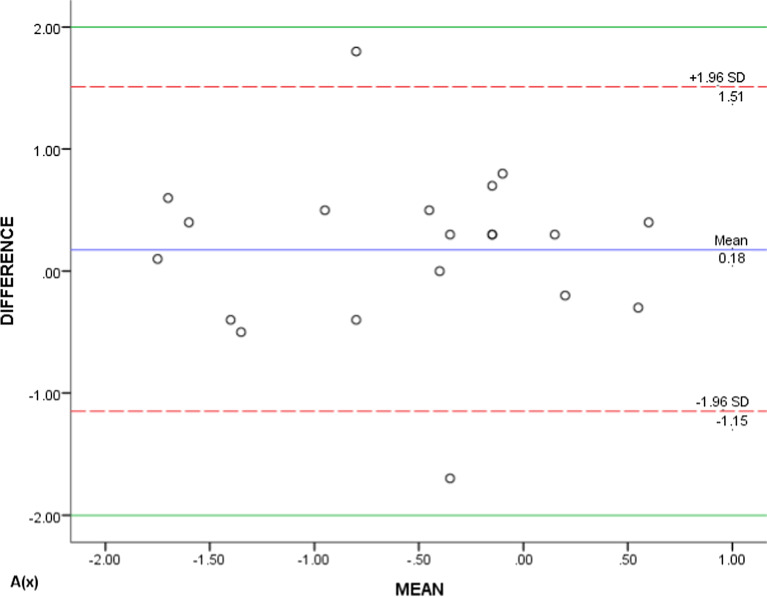
Bland–Altman scatter plot of A(x)

**Fig. 8:**
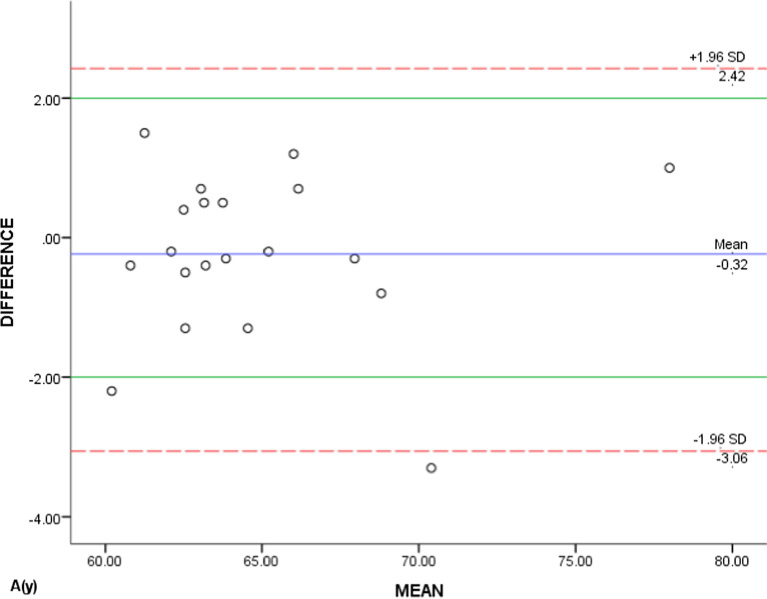
Bland–Altman scatter plot of A(y)

**Fig. 9:**
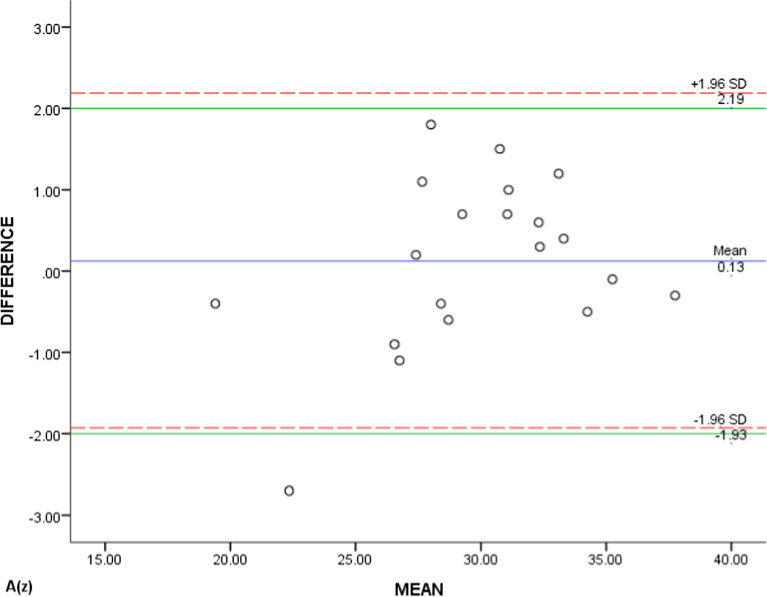
Bland–Altman scatter plot of A(z)

Due to the large amount of data, only the scatter plots of A(x), A(y) and A(z) generated by the Bland–Altman method were showed representatively (Figs. 7, 8, 9). The majority (90%, 95%, and 95%) were scattered within the range of Mean(d) ± 1.96Sd, that is, 95% limits of agreement (95% LoA), and also within the clinical relevance of 2 mm (100%, 90%, and 95%), which could be concluded that the T0 and T1 3D coordinates of ‘A’ were of clinically acceptable agreement. Same results were obtained from the measurements of the rest landmarks.

As for deviation analysis, the overall mean RMSD was 1.85 ± 0.10 mm, within the clinical relevance of 2 mm (Table 4). VSP acquired good accuracy in this study. There was no significant difference between the two means of operations (Table 5). Operation A: Segmental LeFort I osteotomy, BSSRO and mandibular anterior subapical osteotomy (with or without genioplasty); Operation B: Segmental LeFort I osteotomy and BSSRO (with or without genioplasty).

**Table 4:** Mean, standard deviation and RMSD of deviation analysis (mm)

		Mean	SD	RMSD
Operation A	1	0.06	1.74	1.75
	2	0.12	1.88	1.89
	3	0.15	1.95	1.96
	4	− 0.25	1.92	1.93
	5	0.16	1.90	1.92
	6	0.20	1.85	1.86
	7	0.19	1.93	1.95
	8	0.16	1.73	1.76
	9	− 0.18	1.76	1.78
Operation B	1	− 0.18	1.96	1.97
	2	0.10	1.83	1.85
	3	0.07	1.70	1.72
	4	0.25	2.00	2.01
	5	− 0.13	1.70	1.72
	6	− 0.21	1.86	1.88
	7	0.09	1.72	1.73
	8	0.19	1.77	1.79
	9	0.14	1.71	1.72
	10	0.24	1.90	1.91
	11	− 0.19	1.92	1.94
Mean of A				1.87 ± 0.08
Mean of B				1.84 ± 0.11
Overall mean				1.85 ± 0.10

**Table 5:** Difference between two means of operations

	Levene test		Independent sample t test		
	F	*P*	t	*P*	Difference of mean
RMSD	1.58	0.22	0.60	0.56	0.03

## Discussion

The two-dimensional lateral cephalometric X-ray was most commonly used in clinical practice, but it was less accurate than CBCT due to reasons such as image quality and overlap [22, 23]. In this study, the 3D locating of landmarks improved the accuracy while ensuring the efficiency. For the reason that segmental osteotomy was included, the landmarks near the osteotomy line such as ANS, PNS, 13, 23, 33, and 43 were added to increase the representativeness of the selected landmarks. Moreover, it was easier to locate on teeth than on bone and was less affected by metal artifacts.

This study compared the 3D coordinates of the landmarks on 3D models of T0 and T1, and specified the positive values, instead of simply comparing the distance to the reference planes. We used this method to avoid ignoring the difference, where for instance equal distances were measured on T0 and T1 as the landmarks were located symmetrically on two sides of the reference plane but the difference was calculated as ‘0’, that is, a "false negative" result. This situation was likely to occur to landmarks near the sagittal plane, such as A, U1, L1, etc. However, the midline of the upper and lower dentition corresponding to U1 and L1 was a clinical issue, so the accuracy of the measurement was more important. The reliability of this study was increased by the method we used.

‘T1-T0’ had both positive and negative values. A positive value meant the landmark of T1 was on the left to that of T0 in the coronal direction, similarly, on the front to that of T0 in the sagittal direction and downside to that of T0 in the vertical direction. Therefore, to evaluate the difference between the overall data of T1 and T0, RMSD needed to be used to keep both positive and negative differences.

Our study had similar results as Giovanni Badiali et al. did [24], the RMSD was slightly larger than that of the studies only involving skeletal class III cases without segmental osteotomy [15, 25]. Possible factors included: 1. The operations involved in our study were more difficult, there were more osteotomy lines, and the range of movement and rotation of bone segments was larger; 2. It was actually impossible to take CBCT immediately after the operation. The instability of the bone segments and the strong muscle strength of skeletal class II cases after the operation might change the position of the landmarks; 3. Comparing coordinates retained more differences that should be retained than comparing distances.

The RMSD in the sagittal direction was larger, which may be due to the large amount of surgical movement, and the difficulty of controlling the position of osteotomy line and amount of bone removal in the tooth extraction area. Moreover, the extraction space sometimes wasn’t completely closed after segmental osteotomy for postoperative orthodontics, which increased the complexity of the operation. Therefore, it was recommended to add the design of osteotomy guiding templates in VSP, so as to control the osteotomy more accurately and increase the accuracy of VSP [26, 27].

Model surgery and VSP cannot simultaneously appear in the evaluation system created by the software, where the stable cranial landmarks were used to establish reference planes to compare the preoperative and postoperative landmarks. In particular, some landmarks of bone could only appear in VSP. Deviation analysis cannot be performed by software, either. Due to individual differences between patients and differences in surgical planning, it was difficult to perform accurate randomized controlled trials with VSP and model surgery. The literature on the accuracy of model surgery mainly involved the traditional orthodontic-orthognathic treatment, while few studies involved SFA and segmental osteotomy. According to the current literature, the accuracy of VSP was similar to or better than that of traditional model surgery [28–31].

The automatic 3D superimposition technology included initial and precise superimposition, where the translation error and rotation error between the point clouds were firstly reduced then minimized. A high-level registration method based on free-form surfaces, that is, iterative closest point (ICP) proposed by Besl et al. [32] could explain the procedure. Kim et al. [33] used software based on this theory to achieve automatic registration in the dental field, with mean deviation of 0.13 ± 0.13 mm; Tang Min [34] compared the accuracy of manual registration and automatic registration of 3D integrated dental models and concluded that the automatic registration method was better, and both methods could establish an accurate 3D integrated model.

The 3D model in this study was composed of about 600,000 triangles. All of the distances between the corresponding point clouds were calculated by computer and the statistical analyses were automatically performed. Theoretically, this method was more accurate and repeatable than manually locating finite landmarks and measuring. However, in the presence of a large number of metal artifacts, the accuracy of the reconstructed 3D models would be affected, and consequently the deviation analysis. There was currently no method to perfectly remove the artifacts of the titanium plates and nails due to its threshold was close to that of the bone, and the resulting error was temporarily inevitable. Because the 3D measuring was less affected by artifacts, our study combined the two methods.

Difference of surgical operations might also influence the accuracy of VSP. The more complicated the operation, the worse the accuracy theoretically. However, patients received the same operation but differed in age, gender, and degree of malocclusion might generate different accuracy, which might be able to explain the result of no significant difference between the RMSD of two surgical operations in this study. Due to the small sample size of different surgical operations, the reliability of the results of separate statistics for different operations would be confirmed after further expansion of the sample size.

In the process of deviation analyses, the red and blue chromatograms were also found appearing in the bilateral mandibular ramus and body, suggesting that the positional change of the proximal bone segment was also one of the factors that needed to be considered in VSP. The above results might help explaining the difference in the overall RMSD of the two measuring methods in this study.

Deviation analysis was suitable for quickly determining whether there were differences between 3D models and where the differences mainly concentrated due to its intuitiveness of chromatogram, but its accuracy was easily affected by the quality of 3D model itself, such as metal artifacts, the compression ratio in reconstruction, and optimizing operation, etc. In addition, the result of the deviation analysis was relatively simple, and the interpretation of local features was not as good as the 3D measuring. On the other hand, 3D measuring alone would not be completely accurate due to the subjectivity of manually locating and the limitation of landmark coverage. The results and conclusions obtained by the two methods in this study were basically the same, which had a certain degree of persuasiveness.

## Conclusions

In conclusion, VSP in segmental osteotomy in combination with bimaxillary orthognathic surgery with SFA proved to acquire accurate outcome in this study. Combined orthodontic-orthognathic treatment could achieve intuitive, accurate and predictable outcomes through VSP.

## Data Availability

The dataset used and/or analyzed during the current study available from the corresponding author on reasonable request.

## Abbreviations

VSP: Virtual surgical planning
SFA: Surgery first approach
3D: Three-dimensional
CBCT: Cone-beam computed tomography
RMSD: Root mean square deviation
CAD/CAM: Computer-aided design and manufacturing
DICOM: Digital imaging and communications in medicine
HP: Horizontal plane
SP: Sagittal plane
CP: Coronal plane
Or_L_: Left orbitale
Or_R_: Right orbitale
P_M_: Midpoint of the left and right porions
N: Nasion
S: Sella point
ANS: Anterior nasal spine
PNS: Posterior nasal spine
A: Subspinale
13, 23, 33, 43: Maxillary canine cusp point
B: Supramental
U1: Contact point of maxillary central incisors
L1: Contact point of mandibular central incisors
16, 26: Mesiobuccal cusp of maxillary first molars
36, 46: Mesiolingual cusp of mandibular first molars
Me: Menton
Pog: Pogonion
BSSRO: Bilateral sagittal split ramus osteotomy
95% LoA: 95% Limits of agreement
ICP: Iterative closest point
